# How to Kill a Craving for Alcohol

**Published:** 1884-12

**Authors:** 


### HOW TO KILL A CRAVING FOR ALCOHOL.

WHILE it is true that many who at one time indulged in ardent spirits have abstained later in life, it is not believed that there is any real cure for the thirst created by alcoholism, but a person that claims to have cured himself gives a remedy that there would be no harm in trying. We reproduce it in the rescued person's own words: "I was one of those unfortunates given to strong drink. When I left it off I felt a horrid want of something I must have or go distracted. I could neither eat, work nor sleep. Explaining my afflction to a man of much education and experience, he advised me to take a dedoction of ground quassia, a half ounce steeped in a pint of vinegar, and to put about a small teaspoonful of it in a little water, and to drink it every time the liquor thirst came on me violently. I found it satisfied the cravings and it also gave a feeling of stimulus and strength. I continued this cure and preserved till the thirst was conquered. For two years I have not tasted liquor, and I have no desire for it. Lately, to try my strength, I have handled and smelt whiskey, but I have no temptation to take it. I give this for the consideration of the unfortunate, several of whom have recovered by means which I no longer require."

**Barley Water for Milk.**—The dilution of cow's milk with water for infants and children is a greivous mistake. It renders the milk less nutritious, thereby making it necessary to give a larger quantity at more frequent intervals, in this way impairing the child's digestive powers. Barley water will act advantageously, but, says the *Medical Age*, much depends on the proper preparation of the barley water. Take an ounce of pearl barley and wash it in cold water, then put it in a vessel containg half a pint of water, and let it be gently heated over the fire, so that the water just simmers a few minutes; now pour off this water, replace it by a pint and a half of water, and boil down to a pint, and you then have barley water.

**About Poisoned Snakes.**—Sir Joseph Fayrer, who has been investing snake-poisoning, says that to him one of the greatest of mysteries is that a poisonous snake cannot poison one of its own species, scarcely its own congeners, and only slightly any venomous snake; but it kills innocent snakes quickly. A vigorous cobra can kill several dogs, or from a dozen to twenty fowls before its bite becomes impotent, and then the immunity is of brief duration, for the virus is rapidly re-secreted.

